# Advances and crosslinking of the piezoelectric nanoplatform: exploring the multifunctionality in different biomedical applications

**DOI:** 10.3389/fchem.2025.1714203

**Published:** 2025-11-25

**Authors:** Jhilik Roy, Muhammad Madni, Amartya Sau, Ruma Basu, Sukhen Das

**Affiliations:** 1 Department of Physics, Jadavpur University, Kolkata, India; 2 School of Science, Harbin Institute of Technology, Shenzhen, Guangdong, China; 3 Department of Physics, Jogamaya Devi College, Kolkata, India

**Keywords:** piezoelectric materials, reactive oxygen species (ROS), biomedical applications, piezodynamic therapy, tissue regeneration, self-powered biosensors

## Abstract

Piezoelectric materials have emerged as versatile platforms with transformative potential in biomedical research, yet their clinical translation remains limited. This mini review examines how these materials generate reactive oxygen species (ROS) under mechanical stimulation to regulate biological processes, enabling antibacterial activity, wound repairing, tissue regeneration, and targeted cancer therapy through piezodynamic, chemodynamic, and photothermal pathways. Beyond treatment, piezoelectric materials facilitate controlled drug and gene delivery and function as self-powered biosensors for real-time monitoring. To this end, we also discuss key challenges hindering clinical translation, including instability, precipitation, fabrication complexity, and long-term biocompatibility, and conclude by outlining future strategies for developing flexible, biodegradable, AI-integrated platforms for precision and adaptive healthcare.

## 1 Introduction

The field of biomedical engineering has witnessed a rapid evolution in recent years, with increasing emphasis on the integration of smart materials that can respond dynamically to biological environments (Chorsi et al., 2019; Deng et al., 2022; Soe et al., 2025). Among these, piezoelectric materials have emerged as a particularly promising class due to their unique ability to convert mechanical stimuli into electrical signals (Ali et al., 2023a; Ali et al., 2023b; Roy et al., 2025a). This electromechanical coupling not only allows for the generation of electrical potentials in response to deformation or stress but also facilitates the initiation of biochemical processes at the cellular and molecular levels. In biomedical research, such functionality has opened new avenues for therapeutic and diagnostic innovations, from promoting tissue regeneration to enabling self-powered sensing systems (Yu et al., 2024a; Wang et al., 2023). Although piezoelectric materials have recently gained attention in biomedical applications for their ability to mediate reactive oxygen species (ROS) generation through piezodynamic therapy (PZDT) (Roy et al., 2025b). This property has positioned them at the intersection of science and technology, supporting a broad spectrum of applications (Chen et al., 2020). Their appeal lies in characteristics such as fast response, high precision, tunable properties, structural diversity, and the capacity to operate across wide frequency ranges (Gao et al., 2025). Despite this progress, the translation of piezoelectric materials into clinical settings remains constrained by several technical and biological challenges, necessitating a comprehensive understanding of their multifunctionality and crosslinking capabilities (Pang et al., 2025; Zhu et al., 2025).

Piezoelectric materials, including ceramics such as barium titanate (BaTiO_3_) and polymers like polyvinylidene fluoride (PVDF), have been widely studied for their ability to generate localized electric fields under mechanical stimulation. These electric fields can influence cell proliferation, differentiation, and communication, making them particularly valuable in tissue engineering and regenerative medicine. More recently, research has revealed that piezoelectric materials can also catalyze the generation of reactive oxygen species (ROS) under mechanical stress, which is known as ‘piezocatalysis’. Reactive oxygen species, when properly controlled, play a critical role in regulating a wide range of biological activities, including antibacterial defense, wound healing, and cancer cell apoptosis (Wang et al., 2025; Yu et al., 2024b). Thus, the piezodynamic effect of these materials introduces a novel therapeutic mechanism that operates without external chemical agents, relying instead on intrinsic material properties and physical stimulation.

In biomedical research, piezoelectric materials hold a distinct advantage, as their properties can be tuned under controlled conditions using external stimuli such as stress, temperature, or applied fields (Ma et al., 2021; Roy et al., 2025b). This ability creates a direct bridge between mechanical processes in the body and electrical and biochemical responses, making them ideal candidates for biomedical applications (Mondal D et al., 2025; Muhammad et al., 2025). Through this, piezoelectric platforms connect material science with biological systems, enabling advances in tissue repair, cellular regulation, localized therapies, diagnostic technologies, and carrying a distinctive therapy named PZDT (L. Wang et al., 2023).

This multifunctional capability has inspired the design of piezoelectric nanoplatforms that integrate various therapeutic modalities. For instance, the synergistic combination of piezodynamic, chemodynamic, and photothermal effects allows for targeted and efficient treatment strategies. In antibacterial applications, mechanically activated piezoelectric nanoparticles can disrupt bacterial membranes and generate ROS, leading to sterilization without antibiotics. Similarly, in cancer therapy, piezoelectric nanomaterials enable site-specific tumor ablation through localized oxidative stress and hyperthermia, minimizing systemic side effects. Moreover, the piezoelectric effect enhances cellular signaling pathways that promote angiogenesis and tissue regeneration, making these materials suitable for wound repair and bone healing. Such cross-disciplinary applications underscore the transformative potential of piezoelectric nanotechnology in the biomedical landscape. In tissue engineering, piezoelectric scaffolds can deliver localized electrical signals that encourage cell growth, differentiation, and regeneration, especially in bone and neural tissues. For wound healing, their piezocatalytic properties help fight bacterial infections, while their ability to release charge carriers supports oxidative stress control and speeds up repair (L. Wang et al., 2023).

Beyond therapeutic uses, piezoelectric materials are also being harnessed for biosensing and controlled drug delivery. Their inherent ability to produce electrical signals in response to physiological motion allows for the development of self-powered biosensors that can continuously monitor biochemical markers such as glucose, pH, and stress hormones. Recently, the development of new piezoelectric nanomaterials has further strengthened their biomedical relevance. Lead-free ceramics, 2D layered structures, and biocompatible composites such as barium titanate (BTO), black phosphorus, and molybdenum disulfide (MoS_2_) are showing excellent dielectric, ferroelectric, and biocompatible properties (Jin et al., 2023; Li et al., 2014; Lipatov et al., 2015). These materials can interact safely with biological systems while maintaining high functional performance, placing them at the center of efforts to design next-generation biomedical devices that are both sustainable and clinically translatable (Yang et al., 2020). The convergence of piezoelectric technology with advanced nanofabrication, bioinformatics, and artificial intelligence further strengthens its role in next-generation medical devices.

However, several challenges continue to hinder the clinical translation of piezoelectric nanoplatforms. Issues such as material instability, nanoparticle aggregation, fabrication complexity, and potential cytotoxicity must be systematically addressed (Ullah et al., 2024a; Yang et al., 2020). Furthermore, achieving long-term biocompatibility and biodegradability remains essential for safe *in vivo* applications. Overcoming these limitations requires interdisciplinary collaboration that bridges materials science, nanotechnology, and biomedical engineering. Future research directions are expected to focus on the development of flexible, stretchable, and bioresorbable piezoelectric materials that can integrate seamlessly with biological tissues. The incorporation of artificial intelligence and machine learning could further optimize the performance of these platforms, enabling adaptive and precision-driven therapies (Roy et al., 2025a).

**FIGURE 1:**
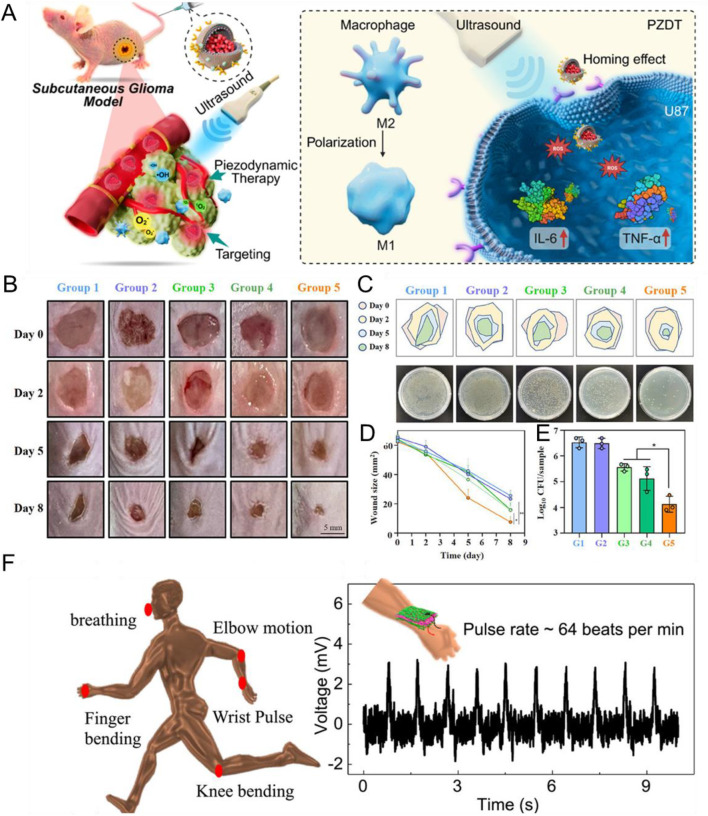
Illustration of the potential of piezoelectric nanocomposites in biomedical applications. **(A)** DGKNO cloaked with U87 glioma cell membrane (CDGKNO) for targeted piezodynamic therapy (PZDT) of subcutaneous glioma. Adapted with permission from Small, copyright © Wiley-VCH. (Ullah et al., 2024b). **(B)** Photographs of MRSA-infected wounds treated with different samples from day 0 to day 8. Scale bar: 5 mm. **(C)** Schematic representation of MRSA-infected wounds and corresponding photographs of MRSA colonies on agar plates under various treatments on days 0, 2, 5, and 8. **(D)** Average infection area growth curves of MRSA-infected mice in different groups (n = 5). **(E)** Colony counts of live MRSA in infected wounds after 8 days of treatment (n = 5, mean ± SD). Panels **(B–E)** Adapted with permission from Advanced Functional Materials, copyright © Wiley-VCH (Dai et al., 2025). **(F)** Schematic overview of human physiological sensing locations and pulse rate monitoring using a piezoelectric sensor. Adapted with permission from Small, copyright © Wiley-VCH (Mondal B et al., 2025).

This mini-review examines the transformative potential of piezoelectric materials in biomedical research, emphasizing their unique properties that modulate biological processes by exploring their multifunctional applications. From dynamic antibacterial therapy and accelerated tissue regeneration to targeted cancer interventions, controlled drug delivery, and self-powered biosensing, this work highlights both the opportunities and the challenges in translating these platforms to clinical practice. Through a critical discussion of mechanistic insights, practical limitations, and emerging strategies, this work sets the stage for translating piezoelectric innovations from the lab to real-world precision medicine and adaptive healthcare. Recent progress in piezoelectric nanocomposites has expanded their biomedical applications, ranging from controlled ROS generation to immune modulation and targeted tumor ablation. Figure 1 demonstrates a representative example, where piezoelectric material DGKNO cloaked with a U87 glioma cell membrane (CDGKNO) enables ultrasound-driven PZDT for precise glioma therapy, underscoring the translational potential of these materials in precision medicine.

## 2 Working mechanisms of piezoelectric effect: production of reactive radicals

The piezoelectric effect is a conversion of mechanical energy into electric energy of certain materials with some structural lattice deformation. Depending on the non-centrosymmetric structure of the piezoelectric material, materials undergo a generation of electrical energy when it is subjected to some mechanical stress (Roy et al., 2024). This phenomenon generally arises from the displacement of the positive and negative charge centers in the lattice, leading to the generation of electric dipoles. These piezoelectric efficiencies are characterized by the Piezoelectric force microscopy (PFM), P-E loops, and some dielectric measurements. These characteristics deeply analyze the ability of polarization, energy conversion efficiency, etc. These are the key factors for generating ROS, determining the multifunctionality of the piezoelectric materials in different biomedical applications (Yuan et al., 2024).

Piezoelectric materials have recently attracted interest for their ability to couple mechanical forces with electrochemical responses (Tu et al., 2020). A key outcome of this process is the generation of reactive oxygen and nitrogen species (ROS and RONS) (Konchekov et al., 2021). These molecules play a dual role in biology: at low levels, they act as essential regulators of cellular function, while at higher levels, they can be harnessed to produce therapeutic effects. ROS include both radical and non-radical forms. Radical species such as superoxide anion, hydroxyl radical, and nitric oxide are highly reactive, while non-radical forms hydrogen peroxide, singlet oxygen, ozone, and hypochlorous acid act as stable intermediates. Under the mechanical stress and electric field, the electrons and holes are separated, and piezo-generated electrons take place in the redox reaction to produce the superoxide radicals, and the holes oxidize water to generate hydroxyl radicals. Different singlet oxygen is produced as a byproduct of the radical formation (Tu et al., 2020; Xu W et al., 2024). The detailed mechanism of ROS generation is depicted through the equations given below.

$$\text{Sample}+\text{Mechanical stress}=e^{-}+h^{+}$$

$$e^{-}+O_{2}=O_{2}^{‐}\;\left(\text{Superoxide Radicals}\right)$$

$$h^{+}+H_{2}O=.OH\;\left(\text{Hydroxyl Radical}\right)$$

In healthy systems, ROS are naturally produced during metabolism in mitochondria, peroxisomes, and the endoplasmic reticulum, as well as by enzymatic activity at the plasma membrane. At physiological concentrations, they serve as short-lived messengers that regulate cell proliferation, differentiation, and immune defense (Ozougwu, 2016). But when overproduced, however, they trigger oxidative stress, damaging DNA, proteins, and lipids, which can lead to apoptosis or other degenerative disorders (Khlyustova et al., 2019; Konchekov et al., 2021).

RONS expand this concept by including nitrogen-derived molecules such as nitrogen dioxide and peroxynitrite. RONS mainly originated from Nitric oxide, commonly reacting with the ROS. Nitric Oxide reacts with the superoxide radical and generates peroxynitrite, and further Oxidation of the Nitric Oxide can easily give a pathway for nitrogen dioxide radicals (Aranda-Rivera et al., 2022).

$$\text{NO}+O_{2}^{-}={ONOO}^{-}\;\left(\text{peroxynitrite}\right)$$

$$\text{NO}+O_{2}={\text{NO}}_{2}\;\left(\text{Nitrogen Dioxide}\right)$$

These compounds are central to immune defense, inflammation, and redox regulation, but their excessive accumulation can also become highly cytotoxic. In piezoelectric materials, ROS and RONS are generated through piezocatalysis (Tu et al., 2020). Mechanical stress polarizes the crystal structure, producing localized electric fields that drive charge separation. Electrons reduce dissolved oxygen to form superoxide, while holes oxidize water molecules to yield hydroxyl radicals (Wang et al., 2022). In the presence of nitrogen compounds, additional reactive nitrogen intermediates can form. This process is similar to photocatalysis but is uniquely driven by mechanical forces such as ultrasound and even natural body movements, making it particularly suitable for biomedical use. Controlled production of ROS and RONS can eliminate drug-resistant bacteria in wounds, modulate signaling pathways for tissue repair, and selectively trigger apoptosis in tumor cells through PZDT (Liu et al., 2023). Clarifying the biological effectiveness of piezoelectric nanoplatforms requires a thorough grasp of the physicochemical basis for ROS and RNOS production mechanisms under piezoelectric stimulation. In addition to controlling radical production, the interaction of band structure modification, charge separation, and interfacial redox reactions also determines the biological processes that these species subsequently trigger. Building on this mechanistic framework, the next section outlines the strategic applications of such reactive oxygen and nitrogen intermediates, along with intrinsic piezoelectric coupling effects, in a variety of biomedical domains, such as tissue engineering, drug and gene delivery, antibacterial therapy, and piezoelectric cancer therapy. By linking mechanical energy to biochemical reactivity, piezoelectric materials provide a powerful and versatile platform for next-generation therapies in wound healing, oncology, and regenerative medicine.

## 3 Biomedical applications of piezoelectric materials

The integration of piezoelectric materials into biomedical research has given entirely new directions for healthcare innovation. To provide a comprehensive overview of recent advancements, various studies employing piezoelectric materials under different external stimuli have been summarized. These investigations include a wide range of biomedical applications, including tissue regeneration, drug delivery, neural modulation, and cardiac repair, where mechanical or ultrasound-induced piezoelectric effects have been utilized to modulate biological responses. An overview of recent piezoelectric materials, their biomedical applications, external stimuli, and key findings from recent studies is summarized in Table 1.

**TABLE 1:** Summary of piezoelectric materials and ultrasound-assisted biomedical applications reported in recent studies.

Material used	Biomedical application	External stimuli	Role of the piezoelectric material	Implication	Key findings	Ref.
BiFeO_3_/PVDF	Antibacterial Therapy	US (10 KHz)	Simultaneous generation of ROS under the external stimuli	Disrupt the bacterial cell without using any antibiotics	>99% degradation of *E faecalis* within 30 min	Chen C et al. (2023)
Fe^2+^ impregnated BiOI	Antibacterial Therapy	US	Generating Reactive oxygen species under ultrasound effect	Bacterial cell rupture and disintegration	>99% degradation of MRSA within 30 min	Roy et al., 2025b
Ce-doped hollow BaTiO_3_	Antibacterial therapy	US (1 MHz)	Successive generation of ROS along with singlet oxygen	Cell disintegration of bacteria by ROS	>90% degradation of MRSA and *P. aeruginosa*	Wei et al. (2023)
BG-KNN	Bone regeneration	US	Generate more ROS for tissue repairing	Cellular proliferation, superior bioactivity	20% BG-KNN maximum bone implants and maintain bioactivity	Mao et al. (2025)
PVDF/p-BaTiO_3_	Bone repairing	US	Provide electrochemical cues to cell	Cell adhesion, proliferation	Scaffold significantly improves the cell response by electric stimulation	Shuai et al. (2020)
SF/PVDF/Mxene	Nerve injury repair	US	Providing higher piezovoltage and antibacterial property	Supplies bioelectric stimulation and antibacterial activity and promotes nerve regeneration	Generate 100 mv piezovoltage and promote axon elongation, myelination	Zhang et al. (2023)
Cu_2-x_O-BaTiO_3_ heterostructure	Piezodynami cancer therapy	US	High-performance singlet oxygen and hydroxyl radicals	Combining ROS production, CDT for invasive cancer treatment	Improvement in cancer therapy by higher ROS and singlet oxygen production	Zhao et al. (2022)
T-BTO with thermosensitive hydrogel	Piezodynami cancer therapy	US	High-performance singlet oxygen and hydroxyl radicals	Offers minimally invasive, biocompatible, localized cancer treatment	Ultrasound enables high ROS production for the cancer cell irradiation with optimised biocompatibility	Zhu et al. (2020)
0.7BiFeO_3_ -0.3BaTiO_3_	Piezodynami cancer therapy	US	PEGylated 0.7BiFeO_3_ -0.3BaTiO_3_ with tuned band structure for ROS generation	Possible engineered band structure for ROS generation and successive therapy through CDT and SDT	Higher production rate of ROS with tuned structure and simultaneous *in vivo*, *in vitro* study for tumour disintegration	Lv et al. (2023)
Hydroxyapatite nanowire PBDF composite	Gene delivery	US	Higher piezoelectric voltage produces cell pores for intracellular delivery	Direct route to deliver the macromolecules to the adherent cells, intracellular payload delivery	Reversible membrane holes and intracellular delivery of FITC-dextran (approximately 75% efficiency) were created by ultrasound treatment. Under certain conditions, HeLa cells exhibited >50% uptake of 40 kDa dextran while maintaining approximately 90% viability	Dolai et al. (2023a)
BTO@hydrogel	Drug delivery	US	Under the ultrasound the key role to the generation of ROS for linkage breaking	Ultrasound-triggered ROS to break the linkages and the successive release of intra-tumoral injection	Generation of a significant amount of ROS for promotes the anticancer drug release to tumour microenvironments	Cui et al. (2025)
BaTiO3-Au	Bio sensing	US	Higher piezoelectric coefficient for ROS production in wireless cell therapy	Shows the targeted bioactivation and biosensing in cancer therapy	High piezoelectric constant (100 p.m./V), higher production of ROS targeting folate receptors in cancer cells	Dolai et al. (2023b)
PZT	Cancer marker sensing	US	High piezoelectric voltage and sensitivity can provide a better detection of cancer	Detection of α-fetoprotein (AFP) and prostate specific antigen (PSA) by altering resonance frequency; high sensitivity ∼0.25 ng/mL; quick detection ∼30 min; small sample sizes ∼1 µL	Rapid diagnostic tool for cancer detection	Su et al. (2017)

### 3.1 Piezoelectric materials for dynamic antibacterial therapy

Antimicrobial resistance (AMR) continues to pose a critical threat to global health, particularly in chronic infections, biofilm-associated medical devices, and drug-resistant pathogens such as methicillin-resistant *Staphylococcus aureus* (MRSA) (He et al., 2024). Piezoelectric materials have emerged as dynamic antibacterial platforms that enhance conventional strategies through mechanical activation, enabling localized and efficient bacterial inactivation and inhibition. Recent advances highlight diverse approaches to exploit piezoelectric effects for antibacterial purposes. Piezo-paint systems, which combine antibiotics with ultrasound treatment, demonstrated remarkable efficacy against *S. Aureus*, *Pseudomonas aeruginosa,* and MRSA biofilms, achieving up to 96% reduction in viable cells (Chen S et al., 2023). Piezoelectric polymer films, including PVDF, PHB, and PVDF-TrFE, have been shown to suppress *Escherichia coli* and MRSA growth under cyclic mechanical stress, benefiting from surface potential generation and enhanced microbial contact (Chen Z et al., 2023). BTO nanoparticles doped with other metals leverage defect engineering to amplify polarization, enabling robust ROS production under ultrasound and effective disruption of both planktonic bacteria and biofilms. Moreover, hybrid nanostructures, including PVDF, poly-L-lactide (PLLA), and PtRu/C_3_N_5_ integrated into hyaluronic acid microneedles, combine piezoelectric stimulation with oxidase-mimic nanozyme activity, achieving near-complete broad-spectrum bacterial killing, *in vitro* and *in vivo,* within minutes of ultrasonic activation (Bhaskar and Han, 2025; Vukomanović et al., 2023; Zheng et al., 2022).

Collectively, these strategies demonstrate the versatility of piezoelectric materials as dynamic antibacterial agents. By combining mechanical responsiveness with tailored material architectures, piezoelectric platforms offer targeted, non-invasive, and highly efficient approaches to combat AMR and MRSA, representing a significant step toward next-generation antimicrobial therapies.

### 3.2 Tissue engineering and regenerative medicine

Piezoelectric materials are rapidly gaining attention in tissue engineering because of their inherent properties of electrical cues and ROS generation that actively guide cell behavior, support extracellular matrix formation, and promote tissue regeneration (Liu et al., 2021). Unlike conventional scaffolds, these materials respond to natural physiological movements, providing a dynamic and precise way to stimulate tissue regeneration (Liu et al., 2021).

In bone repairing, piezoelectric scaffolds made from BTO, potassium sodium niobate (KNN), and their doped derivatives have shown excellent ability to enhance bone formation (Wei et al., 2024). By embedding nanoscale piezoelectric domains into porous structures, mechanical forces and ultrasonic stimulation generate small electrical currents that encourage osteoblast proliferation, differentiation, and mineral deposition. Some scaffolds also incorporate antibacterial features, reducing the risk of infection while supporting robust bone regeneration, an especially valuable trait for orthopedic implants (Jarkov et al., 2022; Shuai et al., 2020). Similarly, for neural tissue engineering, piezoelectric hydrogels, nanofibers, and composite conduits made from materials like PVDF, PLLA, and BTO nanoparticles mimic the natural movements of the body into electrical signals (Kapat et al., 2020; Zhang C et al., 2024). Stimulation from ultrasound or body movements can boost neurite outgrowth, guide axonal alignment, and fine-tune synaptic activity, accelerating recovery in peripheral nerve injuries. Some advanced designs even combine electrical stimulation with growth factor release and nanozyme activity, producing synergistic effects that further support nerve regeneration (Pinho, 2023).

In cardiovascular tissue repair, piezoelectric patches and nanocomposite scaffolds provide electrical cues that synchronize with the heartbeat. Materials such as PVDF-TrFE and BTO nanostructures generate potentials under cyclic strain, enhancing cardiomyocyte proliferation, alignment, contractility, and vascular network formation (Doustvandi et al., 2024). Multifunctional systems that integrate conductive polymers or growth factor-loaded nanoparticles offer additional support for repairing damaged myocardium after infarction (Ye et al., 2025). Moreover, Cinquino et al. highlight the potential of piezoelectric technology in cardiovascular care. Flexible, biocompatible piezoelectric sensors enable noninvasive, real-time monitoring of multiple cardiovascular parameters that demonstrate safety, multifunctionality, and promise for personalized diagnostics and therapeutic interventions in heart disease (Cinquino et al., 2025). Another recent study conveys those piezoelectric patches with the generation of electric charges upon deformation, enhancing the electrical recovery and reducing myocardial infarction. In both the mouse and pig models, the piezo patches are electrically safe and effective in preserving the myocardial integrity and preventing ventricular dilation. These promising piezo patches is a novel therapeutic approach for restoring cardiac electrical and structural function (Monteiro et al., 2025).

Chronic wounds remain a major clinical challenge due to persistent bacterial infection, impaired vascularization, and delayed tissue remodeling (Falanga et al., 2022). Piezoelectric materials offer unique opportunities for wound management by integrating antibacterial effects with regenerative stimulation, providing a dual approach that accelerates healing (Yue et al., 2025). It is essential to note that piezocatalytic activity continues to play a crucial role in wound healing (Ren et al., 2024). Moreover, piezoelectric materials accelerate wound healing through direct electrical stimulation. Endogenous bioelectric signals are known to regulate keratinocyte migration, fibroblast proliferation, and angiogenesis by different pathways (Ali et al., 2023a; X. Wang et al., 2025). These multifunctional systems enable real-time feedback and adaptive therapy without the need for external power sources (Ali et al., 2023b).

### 3.3 Piezodynamic cancer therapy

Piezoelectric materials are highly versatile tools in cancer treatment by coupling mechanical stimulation with catalytic and electrical effects to selectively damage tumor cells (Zheng H et al., 2024). In PZDT, ultrasound or natural body movements activate these materials to produce localized electric fields and ROS, which together disturb redox balance, create oxidative stress, and trigger programmed cell death in cancer cells, while largely sparing healthy tissue (Muhammad et al., 2025). Unlike conventional light-based therapies, PZDT is not limited by penetration depth, making it a promising strategy for treating tumors located deep within the body (Huang et al., 2025).

Their true potential becomes even more evident when piezoelectric systems are combined with other therapeutic approaches. For example, coupling them with chemodynamic therapy (CDT) enhances Fenton and Fenton-like reactions by improving charge separation and lowering the energy barrier for H_2_O_2_ decomposition (He et al., 2025). This results in a higher yield of ·OH, one of the most potent ROS for tumor destruction. Similarly, pairing piezoelectric platforms with photothermal therapy (PTT) allows heat-driven tumor ablation to work hand-in-hand with ROS-mediated apoptosis, producing a strong synergistic effect. Such combinations not only improve treatment precision but also reduce the required doses, lowering side effects and systemic toxicity (Y. Wang et al., 2023).

An exciting development in this field is the creation of Near Infrared-II (NIR-II) guided piezoelectric nanoplatforms. Where 1,000–1,600 nm light is used as the biological window for the deeper tissue analysis and piezo, photodynamic therapy, and different biomedical imaging. These advanced systems are designed to emit in the NIR-II window, which offers deeper tissue penetration, reduced scattering, and sharper resolution (Ullah et al., 2024c; Zhang Z et al., 2024). Along with MRI-guided PZDT, it also plays a pivotal role in the eradication of tumors (Shi et al., 2020). This makes it possible to visualize tumors with high accuracy while simultaneously activating therapy on demand. By merging imaging and treatment in one platform, these nanomaterials allow real-time monitoring of therapeutic outcomes and enable more precise, minimally invasive interventions (Xu H et al., 2024). Therefore, piezoelectric materials are shaping the future of cancer therapy by acting as self-responsive, synergistic, and image-guided systems. Their ability to combine catalytic activity, electrical cues, and advanced optical imaging points toward a new era of precision oncology and next-generation theranostic technologies.

### 3.4 Drug and gene delivery via piezoelectric stimulus

Piezoelectric materials are being increasingly recognized as smart carriers for drug and gene delivery, offering spatiotemporal control in response to ultrasound or mechanical forces with precise, localized effects (Wei et al., 2025). Acting as on-demand nano transducers, they convert external stimulation into electrical signals that reshape carrier surfaces, modulate charges, and trigger-controlled release of therapeutic agents exactly where they are needed (Giorgio et al., 2015). For example, in drug delivery, piezoelectric nanoparticles and nanofibers have been designed to release small molecules in either pulsatile bursts or sustained patterns, offering dosing precision directly at disease sites. Recently, nanofibers made from PVDF-TrFE have responded strongly to ultrasound, accelerating drug release and enabling deeper tissue penetration (F. Wang et al., 2025). By confining release to targeted regions, these systems reduce systemic side effects, a feature particularly advantageous in cancer therapy and inflammatory disorders. The applications extend beyond small-molecule drugs (Vijayakanth et al., 2023). Piezoelectric scaffolds and microneedle arrays are being tailored for nucleic acid and gene delivery. The localized electric fields they generate can gently open cell membranes, enhancing the uptake of plasmids, siRNA, and even CRISPR components without relying on invasive high-voltage methods (Tripathi and Dubey, 2024). This approach not only protects cells from damage but also improves transfection efficiency in tissues that are otherwise difficult to access. Moreover, coupling piezoelectric materials with microelectromechanical systems (MEMS) is further advancing the field (Will-Cole et al., 2022). Concepts such as piezoelectric micropumps for programmable injections or self-regulating microneedles for transdermal therapy present the potential of hybrid systems that unite sensing, feedback, and controlled release into a single platform (Prausnitz, 2004).

In conclusion, these innovations establish piezoelectric carriers as versatile tools for precision medicine. By acting as active participants in therapy, piezoelectric carriers transform drug and gene delivery from passive systems into mechano-responsive platforms that integrate with biomedical devices, adapt to patient needs, and redefine precision in modern nanomedicine.

### 3.5 Piezoelectric biosensing and bioelectronics

By directly translating the subtle motions and biochemical signals of the body into measurable electrical outputs, piezoelectric materials enable continuous, self-sustaining monitoring of physiological activity without relying on external power sources (Zhuang et al., 2025). This capability underpins wearable and implantable devices that track vital signs, tissue mechanics, and biochemical markers in real time, providing a foundation for personalized healthcare and early disease detection (Wu et al., 2025). Wearable piezoelectric sensors employ flexible, stretchable, and biocompatible materials, often designed with kirigami patterns, buckled structures, and serpentine meshes to maintain conformal contact with skin. These systems capture mechanical cues such as heartbeat, respiration, gait, or muscle movement, and chemical cues like sweat metabolites, translating them into electrical signals (Wu et al., 2025). Heart rate variability, pulse waveforms, and breathing patterns can indicate cardiovascular or pulmonary conditions, while continuous monitoring of glucose and other analytes in sweat informs metabolic health. The ability to detect multiple physiological patterns simultaneously allows piezoelectric sensors to construct real-time disease models, enabling predictive health analytics and personalized interventions (Mondal B et al., 2025).

A low-cost, Biocompatible, and non-invasive piezoelectric sensor has been developed for real-time cardiovascular monitoring. The sensor uses a thin ammonium nitride film upon the Kapton for analyzing the heart rate, blood pressure wave, and pulse wave velocity (Cinquino et al., 2025). Furthermore, implantable piezoelectric systems extend these capabilities to internal organs and therapeutic devices. Biodegradable force sensors, piezoelectric patches, and microneedle arrays can monitor internal pressures, provide localized electrical stimulation, and regulate pacemaker activity (Mondal B et al., 2025; Nguyen et al., 2019). These acoustically powered implants can function deep within tissues, while integrated closed-loop feedback enables responsive therapy based on the detected physiological patterns, such as modulating cardiac pacing and drug release. Therefore, by harnessing subtle mechanical and chemical cues from the body, piezoelectric biosensors bridge sensing, monitoring, and therapeutic response (Cheng et al., 2025). Thus, these biosensors go beyond simply converting body movements and biochemical signals into electrical outputs and can detect subtle patterns that indicate early signs of disease (Zhang et al., 2025). By seamlessly integrating continuous monitoring, real-time analysis, and responsive feedback, these devices transform everyday physiological data into meaningful health insights, enabling proactive, personalized care and smarter management of wellbeing.

## 4 Current challenges and outlook

Despite their transformative potential, piezoelectric materials face practical and biological challenges that hinder clinical adoption. A major concern is their stability in aqueous and physiological environments. Many nanostructured ceramics and composites are poorly soluble, prone to aggregation, or susceptible to surface erosion, which reduces bioavailability, polarization, and piezocatalytic efficiency. Such instability compromises applications in wound healing, drug delivery, and implantable devices, where prolonged activity is critical. Mechanical and enzymatic stresses further accelerate degradation, limiting durability under the dynamic conditions of the body. Scalability and cost also remain formidable barriers. High-quality piezoelectric nanocrystals, doped ceramics, and thin films require multistep syntheses, precise doping, and complex post-processing, driving up costs and complicating large-scale fabrication. This restricts their use in resource-intensive applications such as chronic wound dressings or implantable bioelectronics.

Recent advancements in biodegradable piezoelectric materials have created new possibilities for transient biomedical devices that can operate efficiently within living organisms and later decompose without causing harm. Such materials address the long-term biocompatibility and removal concerns. Although their integration in the biological system is very challenging and their degradation kinetics raise concerns about maintaining their piezoelectric property during the operational period. Future research should concentrate on optimizing material composition and microstructure. Notably, Liu *et al.* and Vannozzi *et al.* offer important new information on fabrication techniques and material design methods for the next-generation of biodegradable piezoelectric systems (Liu et al., 2023; Vannozzi et al., 2025).

Another underexplored challenge is long-term safety. While short-term cytotoxicity is often low, chronic exposure to degradation products or residual nanoparticles could induce inflammation, oxidative stress, or immune responses. The potential accumulation of non-degradable fragments raises additional regulatory concerns.

Future progress requires materials that integrate stability, scalability, and biocompatibility. Promising strategies include hydrophilic surface modifications, biodegradable polymer-ceramic hybrids, and organic piezoelectric systems with sustained electromechanical activity. Alongside materials innovation, bridging the gap between *in vitro* and *in vivo* performance is critical, as many candidates fail under real biological stresses.

Looking ahead, the convergence of piezoelectric platforms with digital health and artificial intelligence offers powerful opportunities. Wearable and implantable piezoelectric biosensors can continuously monitor physiological signals and feed real-time data into AI-driven diagnostic systems. Such adaptive, scalable, and biocompatible piezoelectric technologies could accelerate translation from laboratory success to clinical reality, reshaping next-generation biomedical applications.

## 5 Conclusion

Piezoelectric materials are emerging as multifunctional platforms that couple mechanical, chemical, and electrical cues to orchestrate complex biological processes. This mini review emphasized their role in antibacterial therapy, wound healing, tissue regeneration, and cancer treatment through piezodynamic effects and synergistic mechanisms. By integrating drug delivery, biosensing, and adaptive therapeutics, they open avenues for minimally invasive and personalized care. In a nutshell, this mini review frames piezoelectric systems as a convergent biomedical paradigm, crosslinking fundamental science with translational applications to advance precision medicine and adaptive healthcare solutions.
